# MOF-derived Bi_2_O_3_@C microrods as negative electrodes for advanced asymmetric supercapacitors†

**DOI:** 10.1039/d0ra01470b

**Published:** 2020-04-06

**Authors:** Xianbo Yu, Jie Sun, Wenna Zhao, Shihang Zhao, Hongmei Chen, Kai Tao, Yaoping Hu, Lei Han

**Affiliations:** State Key Laboratory Base of Novel Functional Materials and Preparation Science, School of Materials Science & Chemical Engineering, Ningbo University Ningbo 315211 China hanlei@nbu.edu.cn; Key Laboratory for Molecular Design and Nutrition Engineering of Ningbo, Ningbo Institute of Technology, Zhejiang University Ningbo Zhejiang 315100 China wnzhao@nit.zju.edu.cn

## Abstract

Bismuth oxide (Bi_2_O_3_) with high specific capacity has emerged as a promising negative electrode material for supercapacitors (SCs). Herein, we propose a facile metal–organic framework (MOF) derived strategy to prepare Bi_2_O_3_ microrods with a carbon coat (Bi_2_O_3_@C). They exhibit ultrahigh specific capacity (1378 C g^−1^ at 0.5 A g^−1^) and excellent cycling stability (93% retention at 4000 cycles) when acting as negative electrode material for advanced asymmetric SCs. The assembled Bi_2_O_3_@C//CoNi-LDH asymmetric supercapacitor device exhibits a high energy density of 49 W h kg^−1^ at a power density of 807 W kg^−1^. The current Bi-MOF-derived strategy would provide valuable insights to prepare Bi-based inorganic nanomaterials for high-performance energy storage technologies and beyond.

## Abstract 2

Bi_2_O_3_ microrods with a carbon coat (Bi_2_O_3_@C) exhibit ultrahigh specific capacity (1378 C g^−1^ at 0.5 A g^−1^) and excellent cycling stability (93% retention at 4000 cycles) as negative electrodes for supercapacitors.

## Graphical abstract

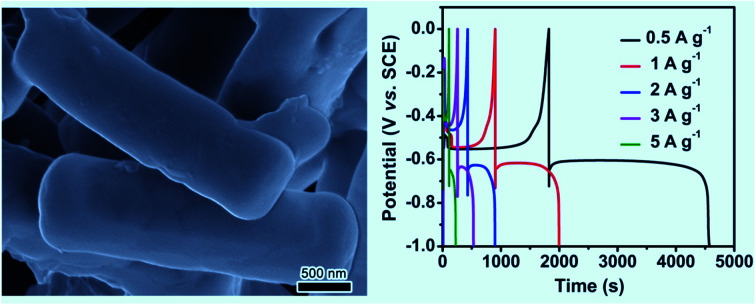

## Introduction

Pollution and depletion of fossil fuels have caused environmental issues and an energy crisis, and it is urgent to develop environmentally friendly and efficient energy storage equipment.^1–3^ Asymmetric supercapacitors (ASCs) have attracted significant and ever-increasing attention as energy storage devices owing to their high power density and long cycle life.^4^ The optimization of electrode materials is intensely investigated to improve the energy density. Carbon-based materials are usually employed as negative electrodes with excellent rate and long lifespan. However, the low specific capacity of carbon materials still restricts the energy density of ASCs for practical applications.^5,6^ Therefore, it is imperative to seek low-cost, high specific capacity and durable negative electrode materials to meet the increasing requirements of peak-power electric vehicles.^7^

Recently, bismuth oxide (Bi_2_O_3_) has been considered as a promising negative electrode material due to its cheaper, environmental friendliness, abundant resources and high theoretical specific capacities (1370 C g^−1^ at 1 A g^−1^).^8,9^ For instance, Qiu *et al.*^10^ synthesized ultrathin Bi_2_O_3_ nanowires by oxidative metal vapor transport deposition technique, which exhibited high specific capacity (576 C g^−1^ at 2 A g^−1^). Shinde *et al.*^11^ grew 3D Bi_2_O_3_ by fast chemical method at room temperature, which demonstrated a specific capacity of 447 C g^−1^ at current density of 2 A g^−1^. Liu *et al.*^12^ designed oxygen-deficient r-Bi_2_O_3_/graphene flexible electrode with high specific capacity of 1137 C g^−1^ at 1 mA cm^−2^. Nevertheless, Bi_2_O_3_ still has disadvantages for ASCs, such as its intrinsically poor electronic and ionic conductivities, the large volume expansion in charging–discharging process. Improved researches have illustrated that the carbon can be used as a buffer layer, which could effectively reduce the morphological change and protect the structure of electrode. The facile design and preparation strategy of Bi_2_O_3_/C composites remains a continuing research to adjust the morphological and electronic structures.^13–16^

Metal–organic frameworks (MOFs) as sacrificial templates to derive nanocarbons or metal compounds/composites are an effective approach to obtain excellent electrode materials with high reversible capacity and cycle performance.^17^ Nano- or micro-structural metal oxides, carbides, phosphides and chalcogenides derived from MOFs have been extensively studied. Especially, the direct formation of carbon-doped composites can improve the conductivity and stability, which can ensure the rapid transfer of electrons.^18^ Ma *et al.*^19^ reported the MOF-derived hybrid Co_3_O_4_/C porous nanowire arrays. To our best knowledge, the fabrication of hybrid Bi_2_O_3_/C derived from Bi-based MOFs has not been reported.^20^

Herein for the first time, we report a Bi_2_O_3_@C microrod through a facile one-step heat treatment, using Bi-based MOF (CAU-17) as both template and precursor. Benefiting from the carbon coated layer from the pyrolysis of CAU-17, it can increase the electrical conductivity and ease the volume collapse during the discharge–charge process. The obtained Bi_2_O_3_@C hybrid as negative electrode for SCs exhibits ultrahigh specific capacity (1378 C g^−1^ at 0.5 A g^−1^) and excellent cycling stability (93% retention at 4000 cycles). In addition, ASC device using a layered double hydroxide (CoNi-LDH) as positive electrode provides a high energy density of 49 W h kg^−1^ at a power density of 807 W kg^−1^.

## Experimental

### Characterizations

Powder X-ray diffraction (PXRD) patterns were implemented by a Bruker AXS D8 Advance diffractometer at 40 kV, 40 mA using a Cu Kα (1.5406 Å) at room temperature. The Fourier transformation infrared spectra (FTIR) were carried out on a NICOLET-6700 infrared spectrometer using the KBr pellet method in the range of 400–4000 cm^−1^. Scanning electron microscopy (SEM) images were obtained from Hitachi S-4800 a field emission scanning electron microscope (FESEM) equipped with an energy dispersive spectrometer (EDS) and mapping operated at an acceleration voltage of 10.0 kV. Transmission electron microscope (TEM) images were recorded using a Thermo Fischer Talos F200× with an accelerating voltage of 200 kV. X-ray photoelectron spectroscopy (XPS) measurements were performed on a Thermo Scientific ESCALAB250-Xi. Thermogravimetric analysis (TG) curve was measured on SII TG/DTA 7300 instrument at a heating rate of 10 °C min^−1^ under N_2_ atmosphere.

### Synthesis of CAU-17 hexagonal prisms

All reagents are commercially produced without further purification. Typically, 715 mg of 1,3,5-benzenetricarboxylic acid and 150 mg of Bi(NO_3_)_3_·5H_2_O were ground uniformly and added to 60 mL methanol. Putting the mixture dissolved under ultrasonic for 10 min. Then homogeneous solution transferring to a Teflon-lined stainless-steel autoclave and heated at 120 °C for 24 h. After cooling down to room temperature, the white powder were collected by filtration and washed with methanol. The final samples were dried at 60 °C for 3 h.

### Synthesis of hierarchical Bi_2_O_3_@C microrods

The obtained CAU-17 hexagonal prisms were annealed to 500 °C for 2 h dwell time under N_2_ atmosphere. The temperature of the tube furnace was increased at a heating rate of 2 °C min^−1^. After cooling to room temperature, the sample of Bi_2_O_3_@C was collected.

### Synthesis of CoNi-LDH sheets

Typically, 2 mmol Co(NO_3_)_2_·6H_2_O, 2 mmol Ni(NO_3_)_2_·6H_2_O and 8 mmol hexamethylenetetramine (HMT) were dissolved in 30 mL distilled water under magnetic stirring to form solution, then the solution was transferred into 50 mL autoclave. The autoclave was sealed and maintained at 100 °C for 10 h, and then cooled naturally. Finally, the products were washed with H_2_O and ethanol, and then dried at 60 °C for 6 h.

### Electrochemical measurements

The electrochemical performance of the tested electrodes was evaluated in a three-electrode system where a saturated calomel electrode (SCE) as the reference electrode and Pt wire counter electrode, respectively, which in alkaline aqueous solution by an electrochemical analyzer system, CHI660E (Chenhua, Shanghai, China). The working electrodes were fabricated from a mixture containing the Bi_2_O_3_@C samples (80 wt%), acetylene black (10 wt%), and polyvinylidene fluoride (PVDF) (10 wt%) with the 1-methyl-2-pyrrolidinone (NMP) to form a slurry. Then, the slurry was coated onto the nickel foam substrates (1.0 cm × 1.0 cm), and dried at 60 °C for over-night, after pressed at 10 MPa for approximately one minute. Cyclic voltammetry (CV), galvanostatic charge–discharge (GCD) and electrochemical impedance spectroscopy (EIS) were used to evaluate the electrochemical performances of the working electrode.

The specific capacity (*Q*_s_, C g^−1^) were calculated by the following equation:1
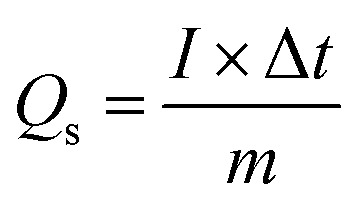
where *I* is the current (A), Δ*t* is the discharge time (s), *m* is the mass of electrode active materials (g).

Before assembling an asymmetric supercapacitor, the load mass of the two working electrodes is balanced by the relationship of charge *q*^+^ = *q*^−^. The energy density (*E*) and power density (*P*) are calculated based on the total mass of the active materials of the two electrodes, according to the following equations:

2 *C* = *Q*_s_/Δ*V*

3
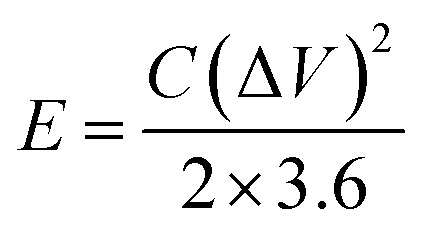
4
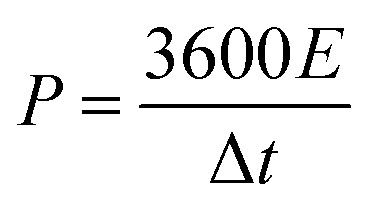
where *V* is the voltage window, Δ*t* is the full discharge time of the assembled ASC devices.

## Results and discussion

**Fig. 1:**
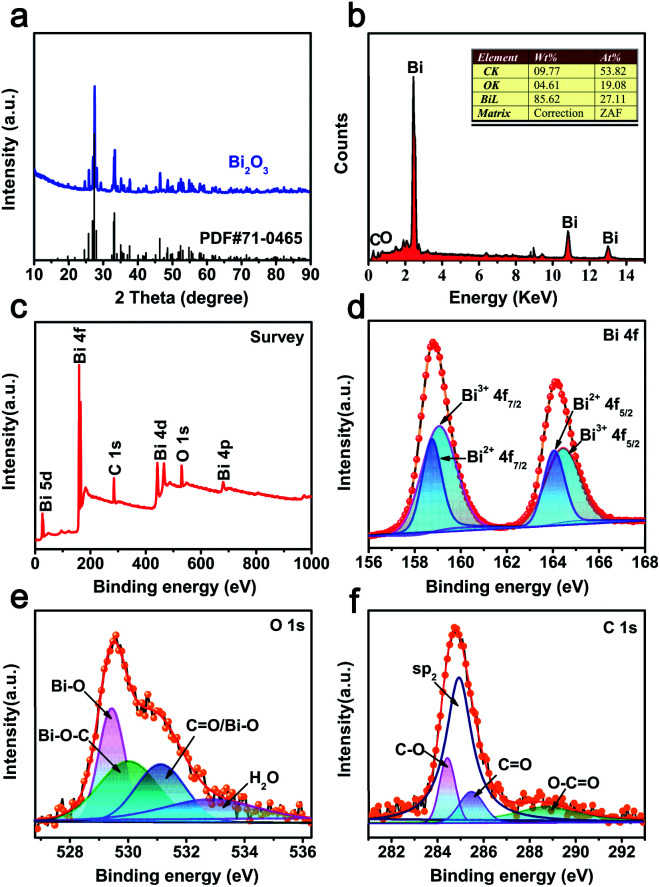
(a) XRD pattern of Bi_2_O_3_@C; (b) EDS spectrum of Bi_2_O_3_@C; (c–f) XPS spectra spectrum of Bi_2_O_3_@C: (c) survey spectra, (d) Bi 4f, (e) O 1s and (f) C 1s peaks.

The CAU-17 MOF precursor was synthesized *via* a solvothermal method according to our previous report.^20^ XRD pattern of the precursor matched well with the phase purity of CAU-17 (Fig. S1a†).^21,22^ The TGA curve in nitrogen atmosphere is show in Fig. S1b.† It can be seen that CAU-17 precursor begins to mass loss at 150 °C, which are attributes to the loss of water and organic molecules (∼12.5 wt%).^22^ The second step at 410 °C corresponds to the decomposition of framework, leading to Bi_2_O_3_ (∼33.7 wt%).^23^ Consequently, Bi_2_O_3_@C was obtained by thermal treatment in nitrogen atmosphere and the heating temperature was optimized to be 500 °C (ESI†). The XRD pattern of (Fig. 1a) indicates that the peaks of 27.7°, 33.5° and 46.4° located at 2*θ* correspond to (012), (022) and (221) planes, respectively, which can be well indexed to monoclinic α-Bi_2_O_3_ phase (JCPDS card no. 71-0465).^24^ The EDS spectrum (Fig. 1b) shows the peaks of Bi, O and C. The FT-IR analysis displays that most of absorption peaks of CAU-17 are disappeared (Fig. S2†), a part of the carbon is retained from the precursor.

XPS test is further carried out to analyse the chemical compositions and detailed surface electronic states. The survey spectrum displays that the existence of Bi, C and O elements in as-prepared materials (Fig. 1c), which are in good match with the EDS results. As shown in Fig. 1d, the spin splitting of Bi 4f peaks consists of Bi 4f_7/2_ and Bi 4f_5/2_, located at 158.8 and 164.1 eV, respectively. Moreover, the peaks can be divided into four peaks, containing two kinds of oxidation states. The higher binding energy peaks of Bi^3+^ are attributed to 159.1 and 164.4 eV, whereas the peaks at 158.7 and 164.0 eV should be designated to Bi^2+^.^25^Fig. 1e shows the O 1s spectrum, the peaks at 529.8 and 530.0 eV correspond to Bi–O and Bi–O–C bonds, 531.1 and 532.9 eV are attributed to the adsorbed water, respectively.^26^ The C 1s profile (Fig. 1f) indicates that the highly intensive peak (∼284.9 eV) can be assigned to sp^2^ hybridized carbon atoms, the other peak at ∼284.4 eV, ∼285.5 eV, and ∼288.7 eV are associated with C–O, C=O, and O–C=O, respectively.^9^

**Fig. 2:**
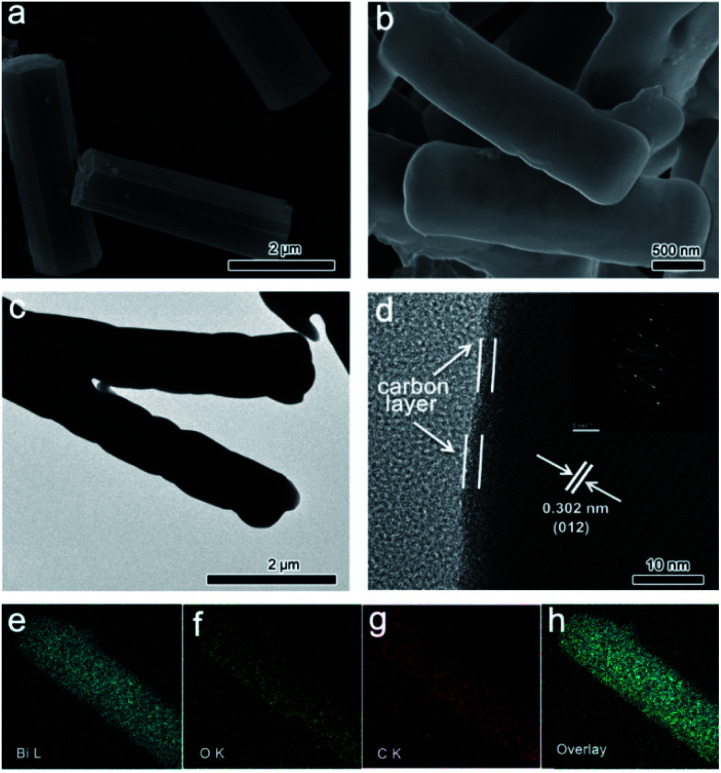
(a) SEM image of CAU-17; (b) SEM, (c) TEM and (d) HRTEM images of Bi_2_O_3_@C; (e–h) The elemental mappings of Bi, O, C and overlay.

The morphology of CAU-17 presents hexagonal microrods (Fig. 2a). SEM image (Fig. 2b) of Bi_2_O_3_@C shows that the microrod morphology remains intact after the pyrolysis, while the hexagonal configuration is damaged. The smooth surface indicates that Bi_2_O_3_ is well encapsulated within the carbon layer. Benefiting from the periodic arrangement of organic motifs and metal nodes in Bi-MOF structure, the carbon formed *in situ* is uniformly coated on the surface of Bi_2_O_3_. Such unique hybrid structure may improve the conductivity and stability, which are beneficial for energy storage. The average diameter and length of Bi_2_O_3_@C microrods are about 820 nm and 3 μm, respectively, which are smaller than CAU-17 precursor (∼1.0 μm and ∼3.5 μm). This size shrinkage could be associated with the massive loss of organic components during the annealing process.^27^ TEM result (Fig. 2c) displays that Bi_2_O_3_@C is dense structure without pores. Furthermore, HRTEM image (Fig. 2d) exhibits the carbon was coated on surface of Bi_2_O_3_. The interplanar spacing between two adjacent lattice fringes is 0.302 nm, which is corresponding to (012) plane of Bi_2_O_3_. Additionally, SAED pattern present a good crystallinity of Bi_2_O_3_. Elemental mappings of an individual Bi_2_O_3_@C microrod indicate all elements are uniformly distributed in the whole structure (Fig. 2e–h).

**Fig. 3:**
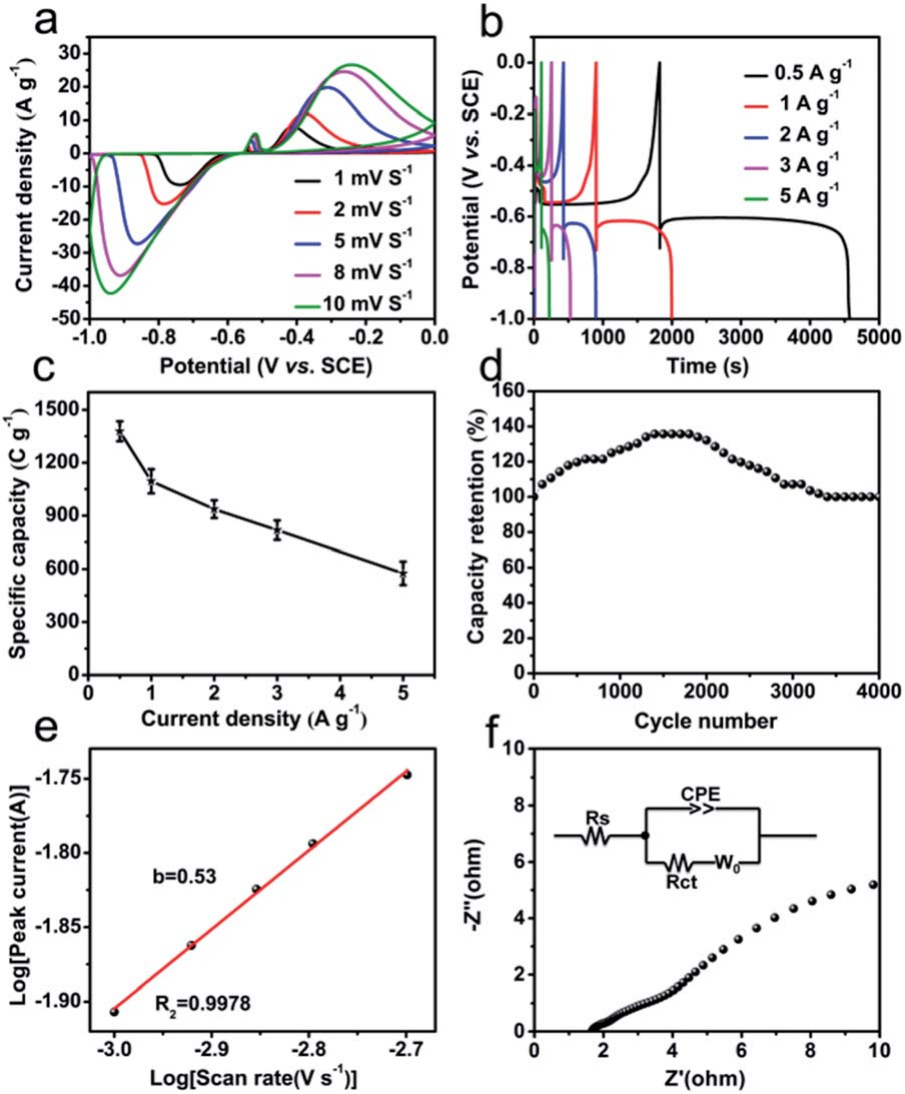
Bi_2_O_3_@C electrode: (a) CV curves at different scan rates; (b) GCD curves at different current densities; (c) the corresponding specific capacities calculated by GCD curves; (d) cycle performance for 4000 cycles at 5 A g^−1^. (e) The separation of the capacitive and diffusion-controlled discharge contributions. (f) Nyquist plots and the equivalent circuit for the EIS fittings.

The electrochemical performances of Bi_2_O_3_@C as negative electrode are evaluated by CV and GCD measurements in 1 M KOH aqueous electrolyte. The CV cures (Fig. 3a) at different scan rates show a reversible charge–discharge response, and the distinct anodic and cathodic peaks are corresponding to the redox reactions of Bi^0+^, Bi^2+^ and Bi^3+^. The possible faradaic reaction mechanism is described as the following equation:^9^ Bi_2_O_3_ + 3H_2_O +6e^−^ ↔ 2Bi + 6OH^−^. Also, the current densities of redox peaks increase with the increased scan rates, indicating fast redox reactions at the electrode/electrolyte interface. The GCD curves of Bi_2_O_3_@C electrode at different current densities (0.5–5 A g^−1^) are revealed in Fig. 3b, the observed plateaus at ∼−0.60 V and ∼−0.55 V, demonstrating the battery-like behaviour and excellent energy storage characteristics. In addition, the specific capacity calculated from GCD tests at different current densities is present in Fig. 3c. The Bi_2_O_3_@C electrode exhibits remarkable specific capacity, which are 1378, 1095, 937, 818 and 575 C g^−1^ at 0.5, 1, 2, 3 and 5 A g^−1^ respectively. The considerable specific capacity is superior to some previously related materials, such as AC (activated carbon)–Bi_2_O_3_ electrode (333 C g^−1^ at 1 A g^−1^),^28^ CQD (carbon quantum dot)–Bi_2_O_3_ (343 C g^−1^ at 0.5 A g^−1^),^29^ mesoporous 3-D Bi_2_O_3_ (447 C g^−1^ at 2 A g^−1^),^11^ Bi_2_O_3_ nanowires (576 C g^−1^ at 2 A g^−1^),^10^ and so on (Table S1†). Moreover, Bi_2_O_3_@C exhibits a remarkable cycling stability. As shown in Fig. 3d, the curve begins to rise gradually, attributing to the activation of electrode. With the increase of cycle number, it finally becomes stable, and remains 93% of the initial capacity at 5 A g^−1^ after 4000 cycles. These superior electrochemical performances of Bi_2_O_3_@C might be attributed to the following reasons. Firstly, the regular structure of CAU-17 could lead to the uniform distribution of the metal components and carbon in Bi_2_O_3_@C microrod, which improves the active surface area and effective electron transport during the electrochemical process. Secondly, the carbon-coated layer could enhance the electrical conductivity and the stability of Bi_2_O_3_@C.^30^

In addition, the charge storage mechanism for Bi_2_O_3_@C electrode is explored by low scan rate of CV, as shown in Fig. 3e. According to the Power law: *i* = *av*^*b*^, where *i* is the current (A), *v* is the scan rate (V s^−1^), *a* is constant and *b* is the Power law exponent. The value of *b* is calculated from the slope of log *i vs.* log *ν* at a constant potential of redox, where *b* = 1 indicates capacitive-controlled mechanism and *b* = 0.5 is characteristic of diffusion-controlled mechanism.^31^ The obtained *b* value of Bi_2_O_3_@C is 0.53 indicates the diffusion charge storage is dominated. The fraction of diffusive charge storage *f*_d_ is determined using the formula: *f*_d_ = (1 − *b*)/0.5, which is used to calculate the capacitive and diffusive contributions of current.^25^ The value of diffusive charge storage is 0.94 for Bi_2_O_3_@C, further demonstrating the diffusion-controlled contribution. There is also a part of the capacitive-controlled contribution, which is caused by the carbon in hybrid Bi_2_O_3_@C. The EIS technique is also employed at the open-circuit potential in the frequency range from 100 kHz to 0.01 Hz. The Nyquist plots of Bi_2_O_3_@C electrode in Fig. 3f show a small line at low-frequency region and a semicircle at high-frequency region. The internal resistance (*R*_s_) is equal to the intercept on the X-axis (1.68 Ω), the charge-transfer resistance (*R*_ct_) corresponds to the diameter of the semicircular loop at the high frequency (0.22 Ω). Moreover, the line with large slope is named Warburg resistance in the low frequency region, suggesting Bi_2_O_3_@C has excellent ion diffusion and fast charge transport speed.

**Fig. 4:**
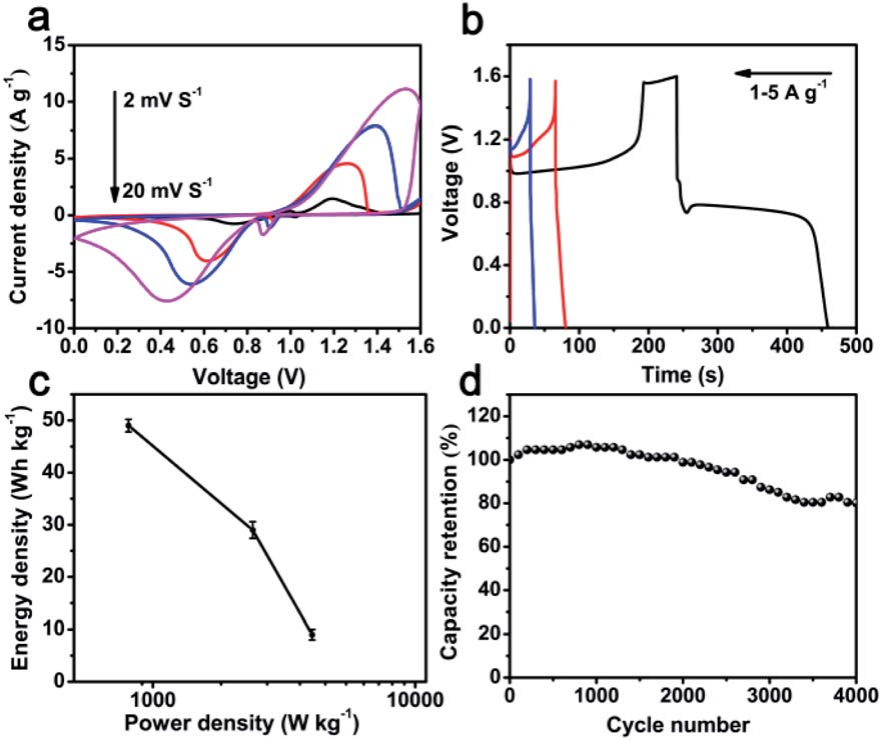
Bi_2_O_3_@C//CoNi-LDH ASC device: (a) comparison of CV curves at different scan rates; (b) GCD curves at different current densities; (c) Ragone plots; (d) cycling performance.

To further investigate the practical application of hybrid Bi_2_O_3_@C electrode, an asymmetric supercapacitor (ASC) device is assembled in 1 M KOH electrolyte by employing Bi_2_O_3_@C/NF (nickel foam) and layered double hydroxide (CoNi-LDH/NF) as negative and positive electrode, respectively. The CoNi-LDH nanoflowers composed of ultrathin nanosheets are prepared through a typical method.^20,32^ The SEM and electrochemical properties of CoNi-LDH are displayed in Fig. S3.† The specific capacities of CoNi-LDH are 566, 544, 517, 459 and 354C g^−1^ at 1, 2, 3, 5 and 8 A g^−1^. The CV curves of ASC device are shown in Fig. 4a, the redox reaction peaks are revealed to different scanning rates (2–20 mV s^−1^) at the voltage range of 0–1.6 V, suggesting fast charge–discharge properties of the ASC device. The GCD curves of ASC device are shown in Fig. 4b. The specific capacity is excellent at voltage plateaus from 0 to 1.6 V, achieved 219 C g^−1^ at the current density of 1 A g^−1^. Ragone plots (energy density *vs.* power density) of ASC device is depicted in Fig. 4c. The energy density of Bi_2_O_3_@C//CoNi-LDH device is 49 W h kg^−1^ at a power density of 807 W kg^−1^, and still maintains 9 W h kg^−1^ at a high power density of 4050 W kg^−1^, which are advantageous over many previously reported ASC, such as AC//Bi_2_O_3_@C (18.94 W h kg^−1^, 1267 W kg^−1^),^33^ ESCNF@Bi_2_O_3_//CF@NiCo_2_O_4_ (25 W h kg^−1^, 786 W kg^−1^),^34^ Bi_2_O_3_//MnO_2_ (9.1 W h kg^−1^, 3370 W kg^−1^),^35^ Bi_2_O_3_–Ni–F//graphite (11 W h kg^−1^, 720 W kg^−1^)^36^ CoNi-LDH//AC (20.38 W h kg^−1^, 800 W kg^−1^).^37^ The capacity retention of Bi_2_O_3_@C//CoNi-LDH ASC is shown in Fig. 4d. It is noted that the cycling performance is unchanged at initial 2000 cycles, and still retains 81% of the initial capacity after 4000 cycles at a current density of 5 A g^−1^, suggesting a favorable stability of this ASC device. These results confirm that as-prepared Bi_2_O_3_@C is expected to be a potential candidate as negative electrode material and satisfy requirements of high-performance ASC device.

## Conclusions

In summary, a facile one-step pyrolysis method is used to synthesize Bi_2_O_3_@C negative electrode material through Bi-MOF-template-directed strategy. Benefiting from the calcined MOF at N_2_ atmosphere, a large amount of carbon is retained in the composite product, increasing the electrical conductivity of Bi_2_O_3_@C composite materials. The Bi_2_O_3_@C as a battery-type negative electrode for SCs exhibits ultrahigh specific capacity of 1378 C g^−1^ at a current density of 0.5 A g^−1^ and excellent cycling stability of 93% retention after 4000 cycles. Moreover, a constructed Bi_2_O_3_@C//CoNi-LDH ASC device exhibits high energy density of 49 W h kg^−1^ at a power density of 807 W kg^−1^. All above-mentioned advantages demonstrate that the current approach of Bi-MOFs-derived strategy would provide valuable insights to prepare Bi-based inorganic nanomaterials for high-performance energy storage technologies and beyond.

## Conflicts of interest

There are no conflicts to declare.

## Supplementary Material

RA-010-D0RA01470B-s001
