# Supplementary material for: MOF-derived Bi2O3@C microrods as negative electrodes for advanced asymmetric supercapacitors
Source: RSC Adv. 2020 Apr 6;10(24):14107–12. doi: 10.1039/d0ra01470b (PMC9051642; doi:10.1039/d0ra01470b)
Supplement: RA-010-D0RA01470B-s001 [file RA-010-D0RA01470B-s001.pdf]

Electronic Supplementary Material (ESI) for RSC Advances.
This journal is © The Royal Society of Chemistry 2020

Supporting information

MOF-Derived Bi_2O_3@C microrods as negative electrodes for advanced asymmetric supercapacitors

Xianbo Yu[a,b], Jie Sun[a], Wen-Na Zhao[b,*], Shihang Zhao[a], Hongmei Chen[a], Kai Tao[a], Yaoping Hu[a] and Lei Han[a*]

[a] State Key Laboratory Base of Novel Functional Materials and Preparation Science, School of Materials Science & Chemical Engineering, Ningbo University, Ningbo 315211, China. E-mail: hanlei@nbu.edu.cn

[b] Key Laboratory for Molecular Design and Nutrition Engineering of Ningbo, Ningbo Institute of Technology, Zhejiang University, Ningbo, Zhejiang 315100,China. E-mail: wnzhao@nit.zju.edu.cn

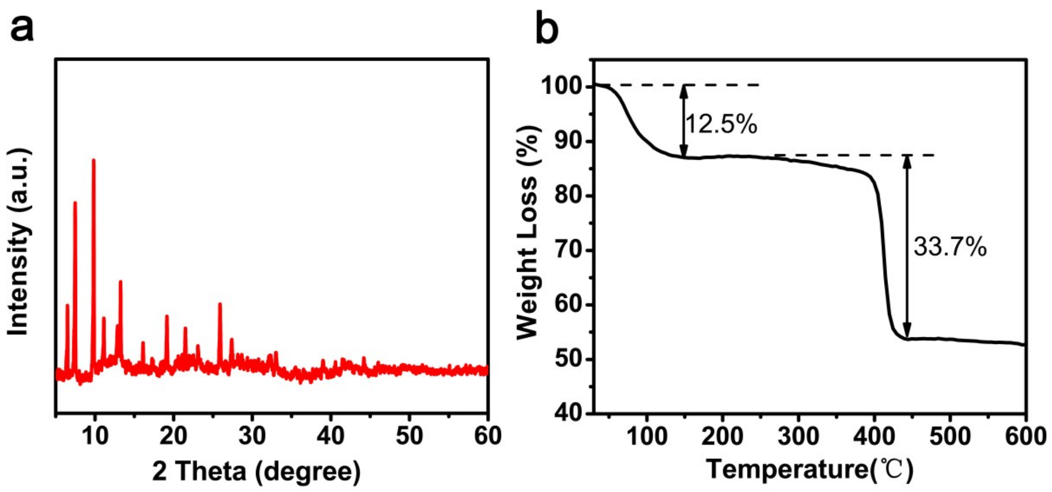

Figure S1. (a) XRD pattern of CAU-17 hexagonal prisms. (b) TGA characterizations of CAU-17.

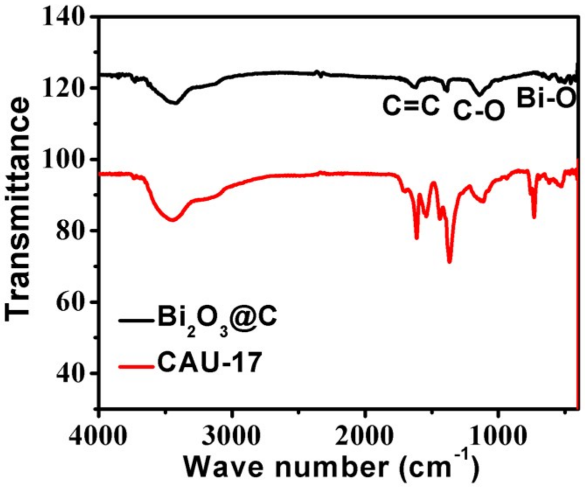

Figure S2. FT-IR spectrum of Bi_2O_3@C and CAU-17.

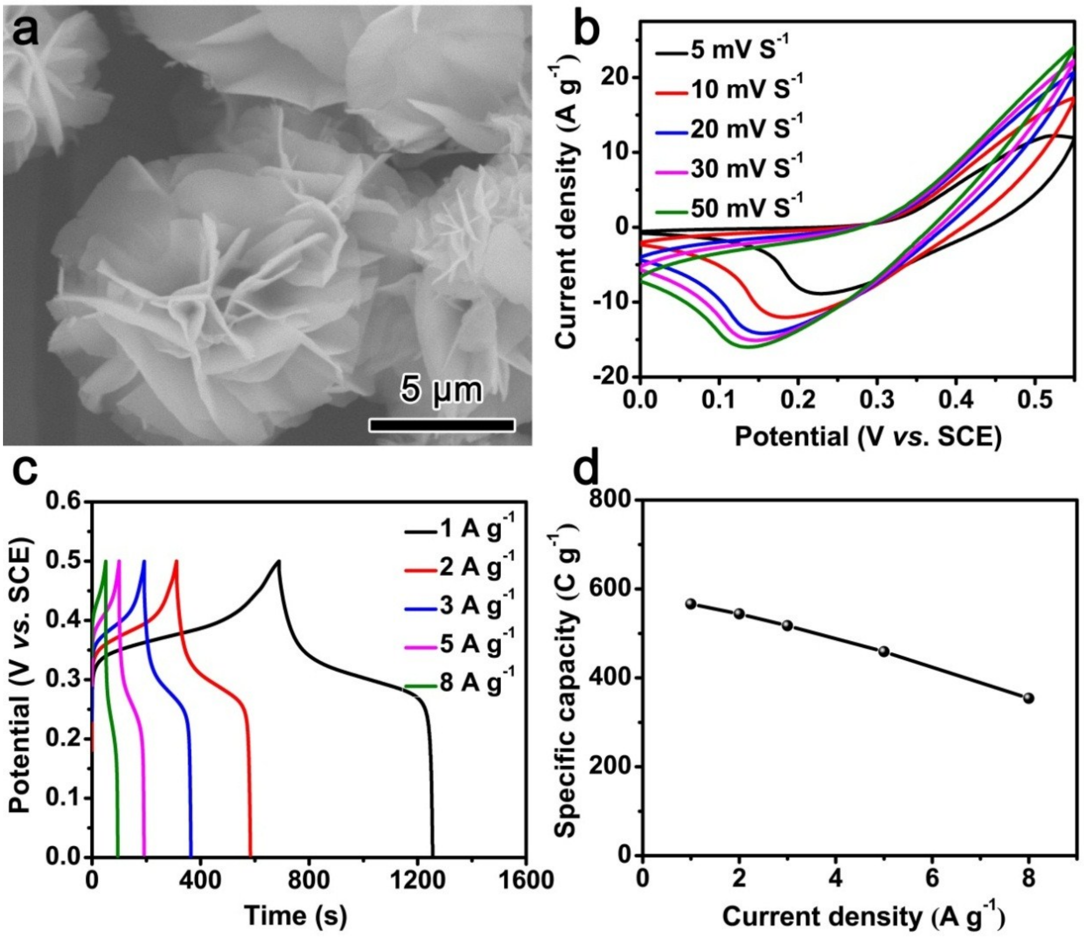

Figure S3. (a) SEM image of CoNi-LDH. (b) CV curves of CoNi-LDH electrode at different scan rates; (c) GCD curves of CoNi-LDH electrode at different current densities. (d) The specific capacity of CoNi-LDH.

Table S1. Electrochemical performance of representative bismuth oxide electrodes

Electrode materials	Specific capacities	Current density	Refs
**Bi_2O_3@C**	745 C g^{-1}	2 A g^{-1}	1
**3-D Bi_2O_3**	447 C g^{-1}	2 A g^{-1}	2
**Bi_2O_3 nanowires**	576 C g^{-1}	2 A g^{-1}	3
**Bi_2O_3**	920 C g^{-1}	2 A g^{-1}	4
**Bi_2O_3@MnO_2**	70.2 C g^{-1}	2 A g^{-1}	5
**β-Bi_2O_3**	783 C g^{-1}	2 A g^{-1}	6
**Bi_2O_3@C**	937 C g^{-1}	2 A g^{-1}	This work

References

1. Z. Zhao, Y. Ye, W. Zhu, L. Xiao, B. Deng and J. Liu, *Chin. Chem. Lett.,* 2018, **29**, 629-632.
2. N. M. Shinde, Q. X. Xia, J. M. Yun, R. S. Mane and K. H. Kim, *ACS Appl. Mater. Interfaces,* 2018, **10**, 11037-11047.
3. Y. Qiu, H. Fan, X. Chang, H. Dang, Q. Luo and Z. Cheng, *Appl. Surf. Sci.,* 2018, **434**, 16-20.
4. H. Su, S. Cao, N. Xia, X. Huang, J. Yan, Q. Liang and D. Yuan, *J. Appl. Electrochem.*, 2014, **44**, 735-740.
5. J. Ma, S. Zhu, Q. Shan, S. Liu, Y. Zhang, F. Dong and H. Liu, *Electrochim. Acta,* 2015, **168**, 97-103.
6. X.-J. Ma, W.-B. Zhang, L.-B. Kong, Y.-C. Luo and L. Kang, *Electrochim. Acta*, 2016, **192**, 45-51.
